# Craspase Protease Activation Is Sensitive to Oncogenic Single-Nucleotide RNA Mismatches

**DOI:** 10.1021/acschembio.6c00241

**Published:** 2026-07-15

**Authors:** Dani Feldmann, Sam P. B. van Beljouw, Anna C. Haagsma, Konstantinos Kalogeropoulos, Aswin Muralidharan, Stan J. J. Brouns

**Affiliations:** † Department of Bionanoscience, 2860 Delft University of Technology, 2629 HZ, Delft, The Netherlands; ‡ Kavli Institute of Nanoscience, 2629 HZ, Delft, The Netherlands; ∥ Department of Biotechnology and Biomedicine, 5205 Technical University of Denmark, DK-2800, Kongens Lyngby, Denmark; ⊥ Center for Translational Protein Design, 5205 Technical University of Denmark, So̷ltofts Plads, DK-2800, Kongens Lyngby, Denmark

## Abstract

The type III-E CRISPR-controlled protease Craspase is distinguished from other type III systems by its single-subunit RNA-guided protein complex and direct coupling of RNA recognition to protease activation without second messenger signaling, making it an attractive development platform for bioengineering and therapeutics. Here, we identify five positions within the CRISPR RNA (crRNA) of Craspase from *Candidatus* “Scalindua brodae” (*Sb*-Craspase) that are sensitive to single-nucleotide mismatches. We leverage these positions to design crRNAs that selectively target clinically relevant single-nucleotide variants (SNVs) in oncogenic RNA transcripts. Using this approach, *Sb*-Craspase is selectively activated by the “undruggable” *KRAS* G12D SNV, while the wild-type transcript does not induce protease activation. Collectively, our results establish a framework for designing crRNAs to target clinically relevant SNVs, laying the groundwork for Craspase-based diagnostics and therapeutics against otherwise intractable oncogenic mutations.

## Graphical abstract

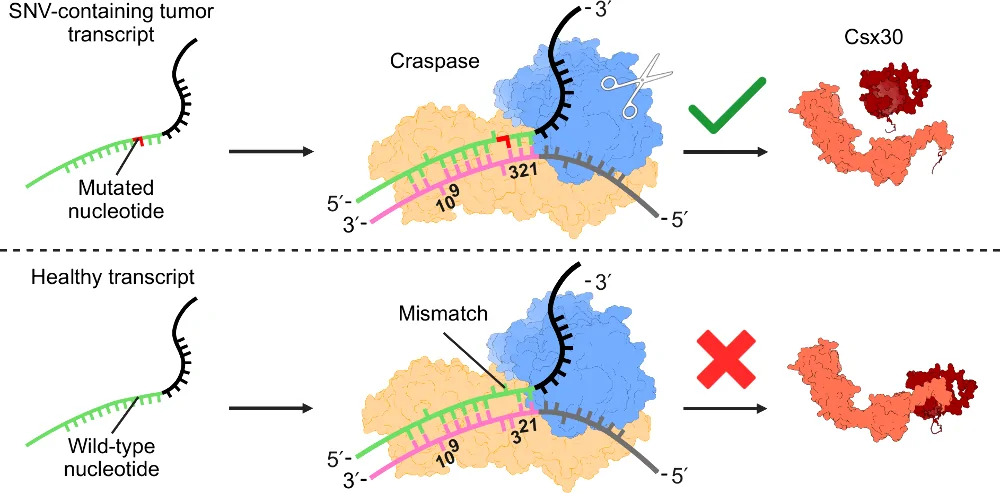

The type III-E CRISPR–Cas system is an RNA-guided immune complex that couples target RNA (tgRNA) recognition to protease activation.^1^ This CRISPR-controlled protease, named Craspase, shows great potential in biotechnological applications, ranging from nucleic acid–based diagnostics^2–4^ to bioengineering,^5^ with emerging potential in therapeutics.^6^ Upon binding of cognate tgRNA to the CRISPR RNA (crRNA), a conformational change is induced in the protease TPR-CHAT (or Csx29), enabling cleavage of the native Csx30 protein substrate.^7–9^ Proteolytic processing of Csx30 triggers downstream antiviral activities, resulting in an abortive infection phenotype.^10,11^

Although not uncommon in type III CRISPR systems,^12–15^ single-nucleotide sensitivity is particularly valuable for applications requiring high selectivity and minimal off-target effects. Single-nucleotide sensitivity refers to the ability of CRISPR–Cas effector complexes to discriminate between perfectly matched and mismatched crRNA–tgRNA duplexes, thereby modulating Cas10 activation and downstream cyclic oligoadenylate (cOA) synthesis. Importantly, such mismatch sensitivity is often position-dependent and is most pronounced near the 5′ end of the crRNA spacer region.^16^

While these observations highlight the promise of mismatch-sensitive RNA recognition, they also raise the question of which CRISPR platforms are best suited to exploit this property in practice. In this context, Craspase presents multiple advantages over conventional RNA-targeting systems. First, its unique single-subunit effector—termed gRAMP (or Cas7–11e)^17^—enables direct relaying of RNA sensing into protease activation without requiring second messengers,^1,8–10^ thereby simplifying the architecture of RNA-responsive sensing circuits. Second, as Craspase is more compact and requires fewer components for protease activation than type III–B complexes, delivery into target tissues for applicational purposes is likely to be more feasible. Third, because Craspase exhibits orthologue-restricted substrate specificity,^18^ multiple Craspase–substrate pairs can operate in parallel without cross-reactivity,^2^ enabling multiplexed diagnostic or therapeutic designs.

Importantly, prior work demonstrated that Craspase can be harnessed for single-nucleotide–level discrimination in a therapeutic context. Here, Craspase from *Desulfonema ishimotonii* was coupled to a Gasdermin D/Csx30 fusion protein to establish an RNA-activated mammalian cell death circuit.^6^ This strategy selectively triggered cell death in virus-infected, senescent and cancer cells expressing single-nucleotide variants (SNVs). Because these cancer-associated SNVs change only a single amino acid relative to wild-type (WT) proteins, they are extremely challenging to target with conventional drugs,^19^ highlighting Craspase as a promising therapeutic candidate. However, it remains an open question whether this level of single-nucleotide resolution represents a general property of Craspase systems, how it varies across orthologues, and how individual positions contribute to target discrimination.

Therefore, we set out to identify which positions in the crRNA of Craspase from *Candidatus* “Scalindua brodae” (*Sb*-Craspase) are sensitive to single-nucleotide mismatches with respect to protease activity. We implemented the first spacer of the CRISPR array (CRISPR1), which has been extensively used to characterize the complex,^1,7,8,11,20^ to monitor Csx30 cleavage in the presence of tgRNAs harboring single mismatches. These mismatches were engineered at positions 1 through 10 starting from the 3′ end of the CRISPR1 tgRNA, and involved bases of the same identity (Figure 1A). Beyond spacer–protospacer complementarity, crRNA design is further constrained by intrinsic self/nonself discrimination mechanisms in native type III antiviral immunity.^21^ Cognate tgRNA triggers ancillary activity, whereas noncognate tgRNA—arising from antisense transcription of the CRISPR array—is distinguished through complementarity between the 5′ repeat of the crRNA and the protospacer flanking sequence (PFS).^8,22^ In the case of noncognate RNA binding, repeat–PFS pairing gives rise to matches that suppress ancillary activation.^8,22^ Consequently, the CRISPR1 tgRNA was designed with a nonmatching PFS (Figure 1A).

**Figure 1:**
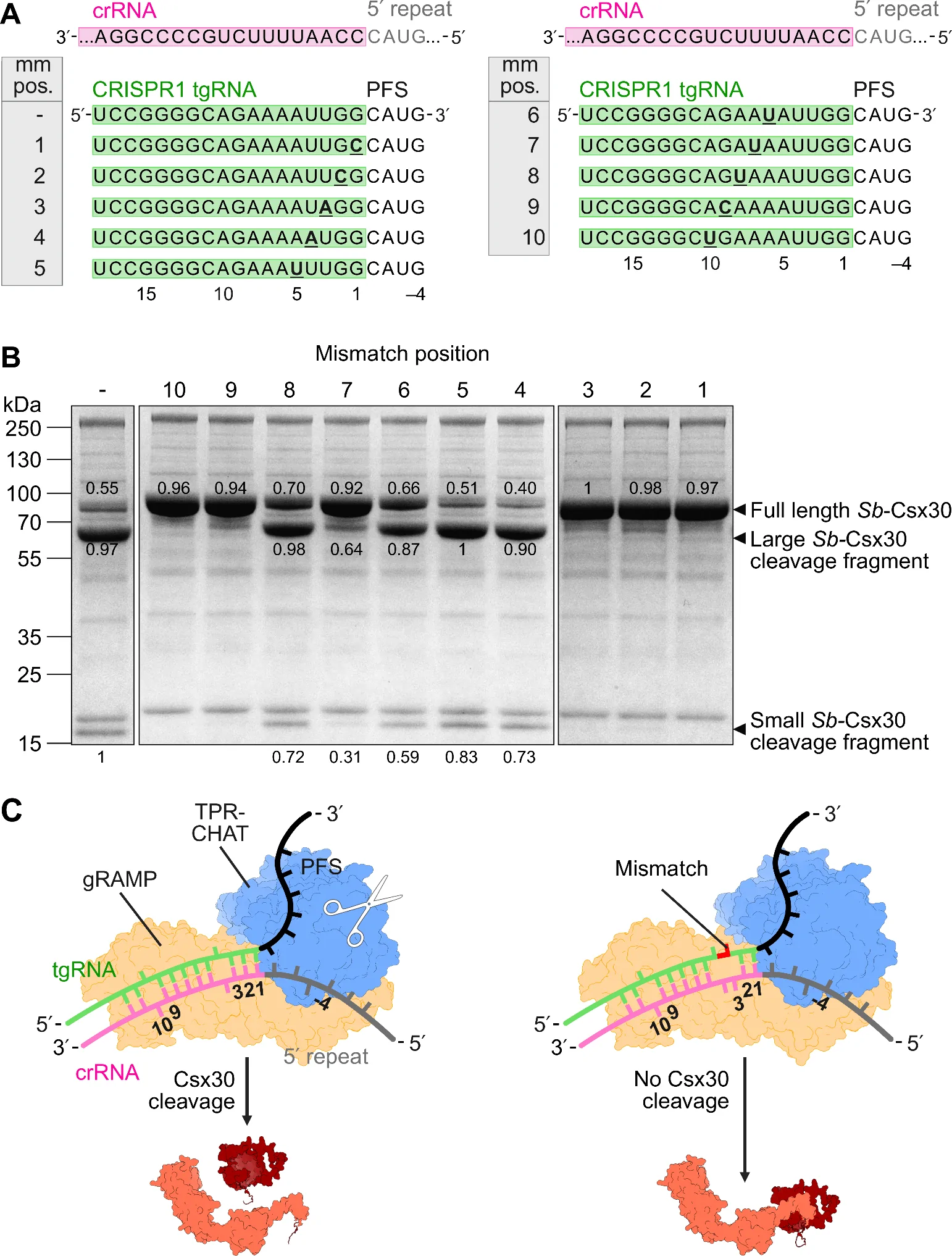
Craspase is sensitive to single-nucleotide mismatches. (A) Single-nucleotide mismatch CRISPR1 target RNAs (tgRNAs) used to identify mismatch sensitive positions (pos.) in the CRISPR RNA (crRNA) of *S. brodae*. PFS, protospacer flanking sequence. (B) SDS-PAGE gel displaying protease activity of *Sb*-Craspase on single-nucleotide mismatch CRISPR1 tgRNA variants. (C) Schematic of protease regulation by *Sb*-Craspase at the single-nucleotide level. Mismatch-sensitive positions in the crRNA spacer portion are highlighted. Positions 4 and 10 in the crRNA are flipped outward.^8^ PDB: 8D9F.

We found that a mismatch at position 1, 2, 3, 9, or 10 prevents Craspase-mediated cleavage of Csx30 (Figure 1B). Surprisingly, position 10 contributed to tgRNA recognition and, consequently, TPR-CHAT regulation. This result is unexpected, as the crRNA and tgRNA nucleotides at this position are oriented away from one another.^8^ Because divalent ions were excluded from our cleavage assays to suppress RNase activity and maintain Craspase in a “stay-on” state, the resulting structure may differ from that reported by Hu et al. Such differences could particularly affect positions 4 and 10, as these divalent ions coordinate residues in their vicinity. In conclusion, *Sb*-Craspase exhibits sensitivity to single-nucleotide mismatches at at least five distinct positions (Figure 1C).

Next, we aimed to exploit this property to selectively induce Craspase protease activity upon recognition of missense SNVs in tumor transcripts. SNVs contribute to the development of a wide range of tumors, including aggressive types such as pancreatic and colorectal cancer with poor treatment options.^23,24^ We selected *KRAS* G12D (c.35G > A), for which no targeted therapies currently exist,^25^ alongside *BRAF* V600E (c.1799T > A), *BRCA2* N372H (c.1114A > C) and *TP53* R175H (c.524G > A), all of which are highly abundant and extensively studied tumor-associated SNVs.^24^ We designed the crRNAs such that they would perfectly match each corresponding SNV-associated tumor transcript, while experiencing a mismatch with the WT variant. Since the first few positions at the 5′ end of the crRNA spacer portion are known to be particularly critical for ancillary activity in canonical type III CRISPR-Cas systems,^15,16^ we restricted mismatch introduction to positions 1, 2, or 3 in the WT transcripts. Moreover, Liu et al. found that single PFS matches at positions −1, −2, or −3 are well-tolerated in *Sb*-Craspase, whereas double matches significantly reduced Csx30 cleavage.^11^ Therefore, we evaluated placing each SNV complement at positions 1, 2, or 3 in the crRNA and selected the position that avoids double PFS matches with the native *S. brodae* 5′ repeat (Figure S1A).

To assess Craspase’s ability to selectively initiate proteolytic activity in response to human tumor-associated SNVs while sparing WT variants, we synthesized 70-nt RNAs carrying *KRAS* G12D, *BRAF* V600E, *BRCA2* N372H, and *TP53* R175H, as well as their WT counterparts. We found that Craspase is only selectively activated in vitro by *KRAS* G12D, cleaving Csx30 while remaining inactive with the WT variant (Figure 2A). In contrast, for *BRAF* and *BRCA2,* we observed Craspase-mediated Csx30 cleavage in the presence of both mutant and WT transcripts. Since neither *TP53* R175H nor the WT variant activated Craspase, we repeated the experiment with modified *TP53* R175H and WT transcripts lacking the −4 PFS match. As expected, TPR-CHAT was able to cleave Csx30, although not in an SNV-sensitive manner (Figure 2B). Interestingly, we observe a greater reduction in Csx30 cleavage upon −4 PFS matching, compared to Liu et al.,^11^ which may be attributable to the additional match at position −5 (Figure S1A).

**Figure 2:**
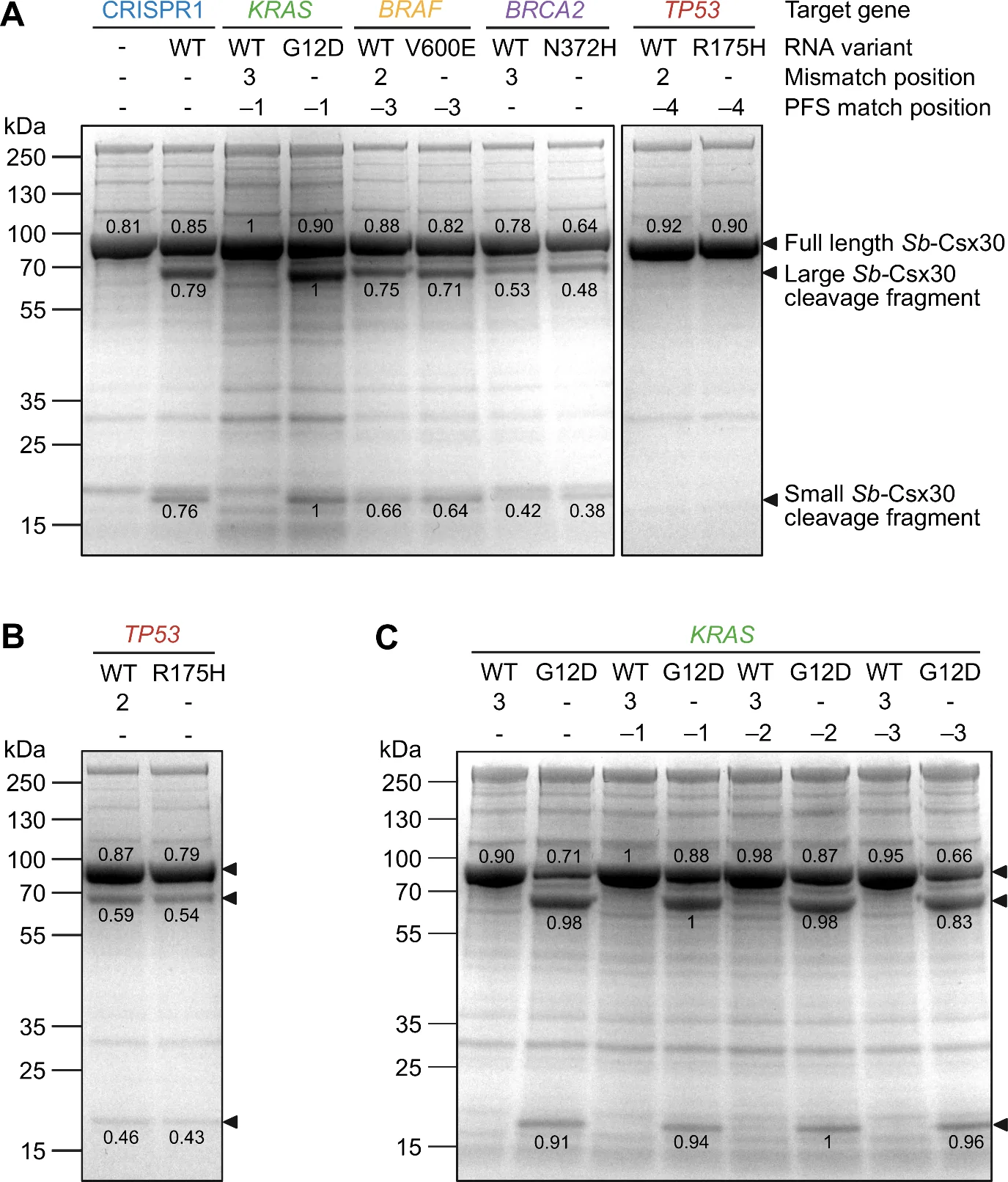
In-vitro analysis of Craspase-mediated Csx30 cleavage in the presence of clinically relevant single-nucleotide variants (SNVs). (A) SDS-PAGE gel displaying in vitro Csx30 cleavage by Craspase in the presence of 70-nt wild-type (WT) and mutant oncogenic target RNAs. Cleavage reactions involving modified PFS nucleotides in *TP53* are displayed in (B), and those for *KRAS* in (C). For each lane, labels indicate the target gene, RNA variant, mismatch position, and protospacer flanking sequence (PFS) match position, listed from top to bottom. Relative cleavage band intensities were normalized to the maximum intensity across panels (A) and (B), and separately for panel (C).

Next, we sought to identify the basis for the observed differences in SNV sensitivity among the selected crRNAs. Previous studies have shown that G:U wobble base pairs can be tolerated in RNA–RNA interactions and may partially preserve target recognition despite sequence mismatches.^26^ However, wobble base pairing alone could not explain the observed activation patterns, since G:U wobble pairing was present in both the *KRAS* WT and *TP53* WT transcripts (Figure S1A), yet only the latter activated Craspase. Furthermore, neither the *BRAF* WT nor *BRCA2* WT transcripts form wobble interactions at the SNV position, yet both are activated despite a mismatch at that site. We also considered whether RNA folding could influence tgRNA binding and thereby explain the absence of Craspase activation by *KRAS* WT. While local secondary structures may obstruct target accessibility, the *TP53* transcripts are predicted to form even more stable secondary structures, with nearly 2-fold lower minimal free energies (MFEs) than those of *KRAS* WT (Figure S1B). Moreover, the predicted MFEs of all tested transcripts are relatively modest and are unlikely to substantially impair RNA targeting.^27^ Collectively, these observations suggest that neither wobble base pairing nor RNA secondary structure can fully explain the observed differences in SNV sensitivity among the investigated tgRNAs.

We then investigated whether PFS matching contributes to these discrepancies. Both the crRNAs targeting *KRAS* G12D and *BRCA2* N372H (crKRAS and crBRCA2, respectively) undergo a mismatch at position 3, but differ in SNV sensitivity. A key difference between these two crRNAs is the presence of a PFS match at −1 for crKRAS, and a PFS free of matches for crBRCA2. Therefore, we hypothesized that only the combination of one mismatch with a single PFS match at a position other than −4 is sufficient to inactivate TPR-CHAT, whereas the single mismatch at position 3 is insufficient on its own. According to this hypothesis, removing the PFS match from crKRAS would abolish its SNV sensitivity. To test this, we systematically moved around the PFS match between crKRAS and its tgRNA by modifying the *KRAS* G12D and WT transcripts. We found that Craspase retains its SNV sensitivity, regardless of PFS match profile (Figure 2C), rejecting our previous hypothesis. Thus, we found single-mismatch SNV sensitivity for *KRAS* G12D. In addition, we show that sensitive mismatch positions are target-specific and must be empirically determined for each new target, rather than following a universal rule.

In conclusion, we demonstrated that Craspase protease activity can be switched on or off, based on single-nucleotide mismatches without following strict universal rules. Using CRISPR1, we identified mismatch-sensitive positions and leveraged them to design crRNAs that selectively target clinically relevant oncogenic SNVs, including the “undruggable” *KRAS* G12D mutation.^25^ However, SNV sensitivity at each tested position was inconsistent across crRNAs, indicating that mismatch position alone is insufficient to predict sensitivity. Our results show that tgRNA sequence context 3′ of the spacer portion also influences activation, consistent with observations in other RNA-targeting CRISPR systems such as *Psp*-Cas13b and *Se*-Csm, where nucleotide identity at specific positions promotes or suppresses activity.^16,28^

In canonical type III CRISPR systems, target recognition is coupled to intrinsic signal amplification via cOA generation. Craspase lacks this amplification; however, the incorporation of a preamplification step could substantially improve sensitivity. Such workflows typically amplify cell-free tumor DNA and subsequently convert the products into RNA for downstream detection. Indeed, while a recent amplification-free Craspase-based sensor achieved a limit of detection of 250 fM,^4^ clinically relevant detection of circulating tumor-derived nucleic acids is generally expected to require sensitivity in the low-attomolar range,^29^ which may be attainable through such preamplification strategies. Importantly, the strengths of a Craspase-based platform are the stability of its protease readout substrates and its multiplexing capability. Four Craspase orthologues have been experimentally characterized to date, highlighting the potential for orthologue-specific target detection within a single assay.^8,9,18,30^ This feature could facilitate simultaneous screening of multiple clinically relevant mutations, such as recurrent *KRAS* mutations at codons 12, 13, and 61,^24^ thereby enabling rapid mutation stratification without the need for comprehensive sequencing workflows. Such an approach could support earlier therapeutic decision-making, which is particularly critical for aggressive cancers driven by *KRAS* SNVs.

Structural studies of Craspase bound to WT and SNV-containing tgRNAs may reveal how single mismatches prevent TPR-CHAT activation and uncover design rules inaccessible to traditional biochemical assays. Unlike type III-A systems, where positions 1 through 7 of the crRNA directly interact with Cas10,^16^ TPR-CHAT does not engage directly with the mismatch-sensitive region of the tgRNA.^8^ It is therefore not immediately obvious how single-nucleotide mismatches lead to TPR-CHAT inactivation. Alternatively, large-scale profiling of protease activity across crRNA–tgRNA pairs could enable identification of mismatch-sensitive crRNAs, for example through consensus spacer analysis, as shown for *Psp*-Cas13b.^28^ In this study, we probed only a single mismatch position per target and focused on the first three spacer positions. Nevertheless, this framework is readily extendable: the experimental strategy shown in Figure 1 can be systematically repeated for any RNA of interest to map SNV sensitivity across positions and spacer designs. Such scalability enables rational identification of mismatch-sensitive crRNAs and demonstrates that precise SNV discrimination is achievable. Together, these results establish Craspase as an SNV-sensitive protease and highlight its potential as a programmable platform for single-nucleotide-resolved RNA sensing.

## Supplementary Material



## Notes

Materials and Methods, supplementary figures, used primers, plasmids and target/CRISPR RNAs (PDF)

## §

Biozentrum, University of Basel, Basel 4056, Switzerland

## #

Division of Infection Diseases & Immunology, Department of Biomolecular Health Sciences, Faculty of Veterinary Medicine, Utrecht University, 3584 CL, Utrecht, The Netherlands.
