# Supplementary material for: Craspase Protease Activation Is Sensitive to Oncogenic Single-Nucleotide RNA Mismatches
Source: ACS Chem Biol. 2026 Jul 15;21(8):1877–82. doi: 10.1021/acschembio.6c00241 (PMC13505182; doi:10.1021/acschembio.6c00241)
Supplement: Supplementary file 1 [file cb6c00241_si_001.pdf]

Supporting information

Craspase Protease Activation Is Sensitive to Oncogenic Single-Nucleotide RNA Mismatches

Dani Feldmann[1,2,†], Sam P. B. van Beljouw[1,2,3,†], Anna C. Haagsma[1,2], Konstantinos Kalogeropoulos[1,2,4,5], Aswin Muralidharan[1,2,6], and Stan J. J. Brouns[1,2,*]

[1]Department of Bionanoscience, Delft University of Technology, 2629 HZ Delft, Netherlands
[2]Kavli Institute of Nanoscience, 2629 HZ Delft, Netherlands
[3]Present address: Biozentrum, University of Basel, Basel 4056, Switzerland
[4]Department of Biotechnology and Biomedicine, Technical University of Denmark, 2800 Kgs. Lyngby, Denmark
[5]Center for Translational Protein Design, Technical University of Denmark, Søltofts Plads, DK-2800, Kongens Lyngby, Denmark
[6]Present address: Virology section, Division of Infection Diseases & Immunology, Department of Biomolecular Health Sciences, Faculty of Veterinary Medicine, Utrecht University, 3584 CL Utrecht, Netherlands

[†]These authors contributed equally.
[*]E-mail: stanbrouns@gmail.com

The PDF file includes:

Materials and Methods
Figure S1
Tables S1 and S2
References

Materials and Methods

Plasmid Cloning

For the construction of plasmids, DNA fragments were amplified using a PCR Thermal Cycler (Biometra GmbH) with the Q5 High-Fidelity Polymerase (New England Biolabs) to introduce appropriate 30-nt overhangs for subsequent DNA assembly. All primers used for PCR are listed in Table S1 and were ordered from Integrated DNA technologies (IDT). 1 µL of DpnI (NEB) was added to each 50 µL PCR reaction mixture, which was subsequently incubated at 37 °C for 1 hour to remove the template DNA.

Fragments were supplemented with a Gel Loading Dye, Purple (6x) (NEB) and run on a 1% (w/v) agarose gel in 1x TAE buffer (Promega) at 100 V for 45 minutes in an electrophoresis apparatus (Advance Company Limited). 1 µL of SYBR Safe DNA Gel Stain (Thermo Fisher Scientific) was added per 10 mL of 1x TAE buffer to visualize the PCR product bands and select for the correct DNA fragments. Bands were cut out and the DNA was extracted using the Zymoclean Gel DNA Recovery Kit (Zymo Research) and cleaned with the DNA Clean and Concentrator Kit (Zymo Research). The plasmids were assembled via HiFi Assembly (NEB) according to the NEBuilder HiFi DNA Assembly Reaction Protocol (NEB #E2621), after which NEB 5-alpha Competent *E. coli* (NEB) were transformed by heat shock at 42 °C using the NEBuilder HiFi DNA Assembly Chemical Transformation Protocol (NEB #E2621). Cells were inoculated overnight on a plate supplemented with LBA and antibiotics corresponding to the antibiotic resistance genes listed in Table S2. Ampicillin was added with a final concentration of 100 µL/mL, streptomycin with 50 µL/mL and chloramphenicol 25 µL/mL. A single colony was picked and grown overnight in 10 mL LB medium containing appropriate antibiotics at 37 °C and 180 rpm. The plasmids were extracted using the GeneJET Plasmid Miniprep Kit (Thermo Fisher Scientific) according to the manufacturer's protocol (#K0502, #K0503).

An overview of all plasmids used in this study is displayed in Table S2. Two plasmids were required for the expression of the Craspase variants. The first plasmid contained an N-terminal Twin Strep-SUMO tagged TPR-CHAT protein from *Candidatus* "Scalindua brodae" (*E. coli* codon-optimized), which is regulated by a LacI repressed T7 promoter (pTPR-CHAT). The second plasmid contained an N-terminal Twin Strep-SUMO tagged gRAMP protein from *S. brodae* (*E. coli* codon-optimized), together with a CRISPR array consisting of five contiguous 36-nt native repeat and 38-nt spacer sequences (pGRAMP-CRISPR-1, pGRAMP-KRAS, pGRAMP-BRAF, pGRAMP-BRCA2 and pGRAMP-TP53). Both gRAMP and the CRISPR array are regulated by a LacI repressed T7 promoter and were assembled using PCR amplification and NEBuilder HiFi

DNA Assembly. pGRAMP-CRISPR-1 contains the first native spacer sequence, whereas pGRAMP-KRAS, pGRAMP-BRAF, pGRAMP-BRCA2 and pGRAMP-TP53 contain a spacer sequence that is derived from the corresponding oncogenic transcript, with the SNV complement located at either the +2 or +3 position of the spacer region. Sequences of the oncogenic transcripts were retrieved from the NCBI database, and the corresponding crRNA sequences were designed accordingly and ordered as gBlocks (IDT). All crRNA and tgRNA sequences used in this study are listed in Table S1. All plasmids were validated using Whole Plasmid Sequencing (Macrogen).

Protein Overexpression and Purification

For the overexpression of different Craspase variants, 20 µL of electrocompetent *E. coli* BL21-AI cells were transformed with a plasmid containing gRAMP and a CRISPR array (pGRAMP-CRISPR-1, pGRAMP-KRAS, pGRAMP-BRAF, pGRAMP-BRCA2 and pGRAMP-TP53) and pTPR-CHAT by electroporation at 2.5 kV, 200 Ω and 25 µF (Thermo Fisher Scientific). Similarly, to overexpress Tag-Csx30, pTag-Csx30 was used to transform 20 µL of *E. coli* BL21-AI cells. Each initial 20 µL cell culture was subsequently supplemented with 1 mL LB and incubated for 1 hour at 37 °C and 350 rpm. Cells were pelleted by centrifugation at 12,000 rpm for 2 minutes at room temperature (Eppendorf 5418 R) and the supernatant was discarded except for ~50-100 µL. The pellets were resuspended and spread over a plate supplemented with LBA and antibiotics corresponding to the antibiotic resistance genes listed in Table S2. Plates were incubated overnight at 37 °C. Protein purification of the Craspase variants and Csx30 was performed according to an earlier described protocol. (1) Csx30 underwent an additional size-exclusion chromatography step, whereas Craspase was purified only via Strep-tag affinity.

SDS-PAGE Analysis

40 µL of protein sample, together with 10 µL Laemmli buffer (375 mM Tris-HCl, 9% (w/v) SDS, 50% (v/v) Glycerol, 0.03% (v/v) Bromophenol blue) and 5 µL of 1 M DTT were incubated at 95 °C for 10 minutes. The samples were loaded on a SurePAGE, BisTris, 10x8, 4-20%, 10 wells (GenScript) gel together with PageRuler Prestained Protein Ladder (10 to 180 kDa) (Thermo Fisher Scientific) and PageRuler Plus Prestained Protein Ladder (10 to 250 kDa) (Thermo Fisher Scientific). Gels were run at 200 V and 500 mA for approximately 40 minutes in a Mini-PROTEAN Tetra Vertical Electrophoresis Cell (Bio-Rad) supplemented with 1x Tris-MOPS-SDS Running

Buffer (GenScript). Gels were washed 3 times for 5 minutes with de-ionized water before staining with Bio-Safe Coomassie G-250 Stain (Bio-Rad) overnight, both under continuous shaking. After de-staining with de-ionized water for 4 hours under continuous shaking, the gels were imaged using the Gel Doc XR+ Imager (Bio-Rad). Relative band intensities were quantified by optical densitometry using ImageJ. (2)

In Vitro Generation and Extraction of 70-nt tgRNA Transcripts

All 22-nt tgRNA transcripts and 90-nt single-stranded (ss) DNA oligos containing the 20-nt T7 promoter sequence were ordered from IDT. Reaction mixtures of 50 µL containing 1x Q5 Reaction Buffer, 1x Q5 High GC Enhancer, 100 µM dNTPs, 2 µM forward primer, 2 µM reverse primer, 0.4 µM of 90 nt ssDNA template and 0.02 units μL^{-1} Q5 High-Fidelity Polymerase underwent the 35 PCR cycles according to the manufacturer's protocol (NEB #M0491), except a 10 s extension and a 20 s final extension time was used. The generated dsDNA oligos were extracted using the protocol described in Plasmid Cloning, except the oligos were run on a 3% (w/v) agarose gel at 70 V for 90 minutes. The templates were subsequently used for *in vitro* RNA transcription using the HiScribe T7 Quick High Yield RNA Synthesis Kit (NEB) according to the manufacturer's Standard RNA Synthesis protocol for RNA products shorter than 0.3 kb (NEB #E2050). The reaction was incubated for 24 hours at 37 °C.

The 70-nt tgRNA samples were supplemented with RNA elution buffer (0.3 M Sodium Acetate pH 5.5 (Thermo Fisher Scientific) and 2% (w/v) SDS) to 200 µL. Then 200 µL Acid-Phenol:Chloroform (Thermo Fisher Scientific) was added to the RNA sample. The mixture was vortexed for 30 seconds and centrifuged for 10 minutes at 13,200 rpm (Eppendorf 5418 R). 160 µL of the liquid from the top portion was subsequently used for ethanol precipitation.

Extraction products underwent ethanol precipitation through the addition of 16 µL 3 M Sodium Acetate pH 5.5 (Thermo Fisher Scientific), 400 µL 100% (v/v) ethanol and 0.8 µL 5 mg mL^{-1} glycogen (Thermo Fisher Scientific) per 160 µL solution. Each total solution was vortexed and kept at –20 °C for at least 30 minutes. The precipitated solution was centrifuged for 20 minutes at 13,200 rpm at 4 °C (Eppendorf 5418 R), after which the supernatant was removed, leaving a pellet with ~50 µL solution. The pellet was then resuspended in 800 µL of 70% (v/v) ethanol and centrifuged for 20 minutes at 13,200 rpm at 4 °C. The supernatant was again removed, leaving a pellet with ~50 µL solution. This wash step was repeated to remove residual salts. The pellets underwent two additional 1-minute rounds of centrifugation at 13,200 rpm and 4 °C to remove any

remaining liquid ethanol. The pellet was dried for 1.5 hours until the ethanol had evaporated and subsequently dissolved in 10 mM RNase-free Tris pH 8.0 (Thermo Fisher Scientific) for at least 30 minutes at 700 rpm and room temperature.

In Vitro Csx30 Cleavage Assay

20 µL of RNase-free reaction mixture containing 0.44 µM Craspase, 2 µM Tag-Csx30, 3 µM tgRNA, 100 mM RNase-free Tris pH 8.0 (Thermo Fisher Scientific), 150 mM RNase-free NaCl (Thermo Fisher Scientific) and 10 mM RNase-free DTT was incubated at 37°C for 1 hour. The reaction mixture was then supplemented with 20 µL of RNase-free water and underwent subsequent SDS-PAGE analysis as described in <u>SDS-PAGE Analysis</u>. All assays were independently performed at least twice using separate protein preparations, and representative gels are shown.

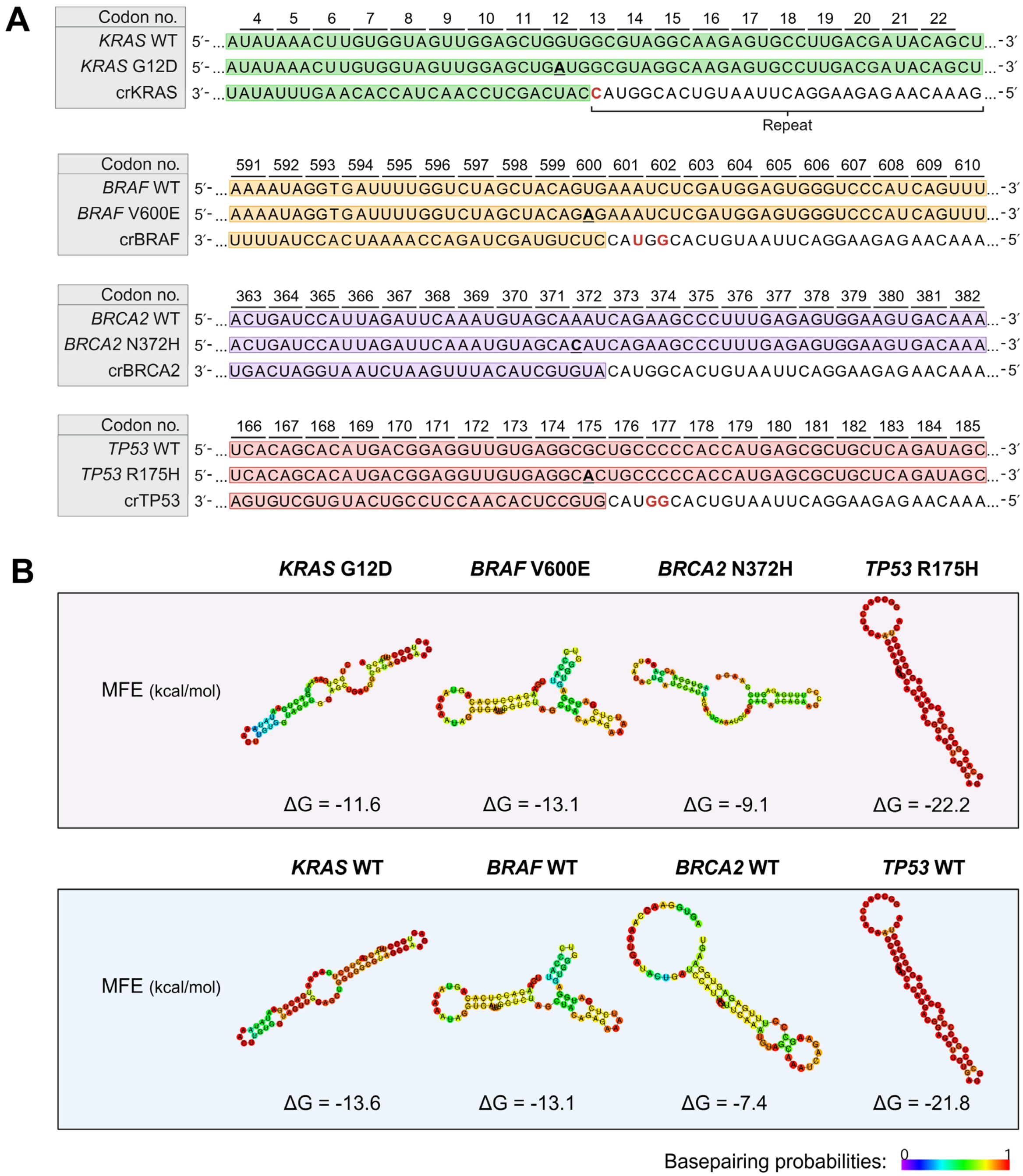

Figure S1: CRISPR RNA design and secondary structure formation of target RNAs. (A) Design of CRISPR RNAs (crRNAs) targeting tumor-associated single-nucleotide variations (SNVs). mRNA sequences of wild-type (WT) and mutant oncogenes along with their corresponding crRNAs. Mutated nucleotides are underlined and shown in bold. Matches between the protospacer flanking sequence (PFS) and repeat within the first five nucleotides of the repeat from the 3′ end are highlighted in red. (B) Minimal free energies (MFEs) and secondary structure base pair probabilities of the oncogenic target RNAs used in this study. (3)

Table S1. Primers, crRNAs and tgRNAs used in this study.

Type	Name	Sequence (5' to 3')	Synthesis	Reference Sequence	Notes
Primer	BN2333	GACGGTATTAGAATTCAAGCTGATCAGAC	IDT	–	Type III-E system Scalindua RAMP repair forward (with BN5965)
Primer	BN2334	GTCTGATCAGCTTGAATTCTAATACCGTC	IDT	–	Type III-E system Scalindua RAMP repair reverse (with BN5964)
Primer	BN2344	TAATACGACTCACTATAGGG	IDT	–	T7 promoter FW primer
Primer	BN2985	TTGTAGGCAATTGCAGCACGATTACCC	IDT	–	Generating gRAMP D698A, rev
Primer	BN2986	CCGGGTAATCGTGCTGCAATTGCCTAC	IDT	–	Generating gRAMP D698A, fwd
Primer	BN3149	TGTGAGCGGATAACAATTCC	IDT	–	Generating backbone of Sb-TPR-CHAT, fwd (with BN6070)
Primer	BN3151	TTTCTACAGGGGAATTGTTATCC	IDT	–	Generating backbone of Sb-TPR-CHAT, rev (with BN6069)
Primer	BN5719	GCCAGAATCGCAATTTTTTCCAGTTTATCG	IDT	–	Generating gRAMP R294A, rev
Primer	BN5720	CGATAAACTGGAAAAAATTGCGATTCTGGC	IDT	–	Generating gRAMP R294A, fwd
Primer	BN5964	TTTTGCTGAAACCTCCGGTAAACCAGCAATAGACATAAGC	IDT	–	13S-S linearize pTU944 backbone with overhangs, fwd
Primer	BN5965	TGTACAATACGATTAGTTTATTGACTACCGGAAGCAGTG	IDT	–	13S-S linearize pTU944 backbone with overhangs, rev
Primer	BN5966	CGGTAGTCAATAAACTAATCGTATTGTACACGGCCGC	IDT	–	Amplify Sb-CRISPR array with 13S-S pTU944 backbone overhangs, fwd
Primer	BN5967	ATTGCTGGTTTACCGGAGGTTTCAGCAAAAAACCCCTC	IDT	–	Amplify Sb-CRISPR array with 13S-S pTU944 backbone overhangs, rev
Primer	BN6117	CTCTTCCTGAATTACAGTGCC	IDT	–	CRISPR1 90 nt template DNA, Rev
Primer	BN6118	ATGGGACCCACTCCATC	IDT	–	BRAF 90 nt template DNA, Rev
Primer	BN6119	ACTTCCACTCTCAAAGGGC	IDT	–	BRCA2 90 nt template DNA, Rev
Primer	BN6120	TCGTCAAGGCACTCTTGC	IDT	–	KRAS 90 nt template DNA, Rev
Primer	BN6121	TGAGCAGCGCTCATGG	IDT	–	TP53 90 nt template DNA, Rev
Primer	BN6215	TGAGCAGCGCTCATGGTGGGGGCAGTGCCTC	IDT	–	-4 mismatch in mut TP53 90 nt template DNA, Rev
Primer	BN6238	TGAGCAGCGCTCATGGTGGGTGCAGCGCCTC	IDT	–	-4 mismatch in WT TP53 90 nt template DNA, Rev
Primer	BN6280	TCGTCAAGGCACTCTTGCCTACGGCATCAGC	IDT	–	-1 mismatch in mut KRAS 90 nt template DNA, Rev

Primer	BN6281	TCGTCAAGGCACTCTTGCCTACAGCATCAGC	IDT	–	-1 mismatch/-2 match in mut KRAS 90 nt template DNA, Rev
Primer	BN6282	TCGTCAAGGCACTCTTGCCTATGGCATCAGC	IDT	–	-1 mismatch/-3 match in mut KRAS 90 nt template DNA, Rev
Primer	BN6283	TCGTCAAGGCACTCTTGCCTACGGCACCAGC	IDT	–	-1 mismatch in WT KRAS 90 nt template DNA, Rev
Primer	BN6284	TCGTCAAGGCACTCTTGCCTACAGCACCAGC	IDT	–	-1 mismatch/-2 match in WT KRAS 90 nt template DNA, Rev
Primer	BN6285	TCGTCAAGGCACTCTTGCCTATGGCACCAGC	IDT	–	-1 mismatch/-3 match in WT KRAS 90 nt template DNA, Rev
crRNA	crCRISPR1	GUUAUGAAACAAGAGAAGGACUUAAUGUCACGGUAC**CCAAUUUUCUGCCCCGGACUCCACGGCUGUUACUAGAG**	IDT (gBlocks)	JRYO01000185.1	Spacer in bold text; repeat in regular text
crRNA	crKRAS	GUUAUGAAACAAGAGAAGGACUUAAUGUCACGGUAC**CAUCAGCUCCAACUACCACAAGUUUAUAUUCAGUCAUU**	IDT (gBlocks)	NM_033360.4	Spacer in bold text; repeat in regular text
crRNA	crBRAF	GUUAUGAAACAAGAGAAGGACUUAAUGUCACGGUAC**CUCUGUAGCUAGACCAAAAUCACCUAUUUUUACUGUGA**	IDT (gBlocks)	NM_004333.6	Spacer in bold text; repeat in regular text
crRNA	crBRCA2	GUUAUGAAACAAGAGAAGGACUUAAUGUCACGGUAC**AUGUGCUACAUUUGAAUCUAAUGGAUCAGUAUCAUUUG**	IDT (gBlocks)	NM_000059.4	Spacer in bold text; repeat in regular text
crRNA	crTP53	GUUAUGAAACAAGAGAAGGACUUAAUGUCACGGUAC**GUGCCUCACAACCUCCGUCAUGUGCUGUGACUGCUUGU**	IDT (gBlocks)	NM_000546.6	Spacer in bold text; repeat in regular text
tgRNA	CRISPR1 WT (22 nt)	UCCGGGGCAGAAAAUUGGCAUG	IDT	JRYO01000185.1	PFS in blue
tgRNA	CRISPR1 +1 mismatch (22 nt)	UCCGGGGCAGAAAAUUG**C**CAUG	IDT	–	PFS in blue; Mismatch position in bold text
tgRNA	CRISPR1 +2 mismatch (22 nt)	UCCGGGGCAGAAAAUU**C**GCAUG	IDT	–	PFS in blue; Mismatch position in bold text
tgRNA	CRISPR1 +3 mismatch (22 nt)	UCCGGGGCAGAAAAU**A**GGCAUG	IDT	–	PFS in blue; Mismatch position in bold text
tgRNA	CRISPR1 +4 mismatch (22 nt)	UCCGGGGCAGAAAA**A**UGGCAUG	IDT	–	PFS in blue; Mismatch position in bold text
tgRNA	CRISPR1 +5 mismatch (22 nt)	UCCGGGGCAGAAA**U**UUGGCAUG	IDT	–	PFS in blue; Mismatch position in bold text
tgRNA	CRISPR1 +6 mismatch (22 nt)	UCCGGGGCAGAA**U**AUUGGCAUG	IDT	–	PFS in blue; Mismatch position in bold text
tgRNA	CRISPR1 +7 mismatch (22 nt)	UCCGGGGCAGA**U**AAUUGGCAUG	IDT	–	PFS in blue; Mismatch position in bold text
tgRNA	CRISPR1 +8 mismatch (22 nt)	UCCGGGGCAG**U**AAAUUGGCAUG	IDT	–	PFS in blue; Mismatch position in bold text

tgRNA	CRISPR1 +9 mismatch (22 nt)	UCCGGGGCA**C**AAAAUUGGCAUG	IDT	–	PFS in blue; Mismatch position in bold text
tgRNA	CRISPR1 +10 mismatch (22 nt)	UCCGGGGC**U**GAAAAUUGGCAUG	IDT	–	PFS in blue; Mismatch position in bold text
tgRNA	CRISPR1 WT (70 nt)	AAGUAUUGCUCUAGUAACAGCCGUGGAGUCCGGGGCAGAAAAUUGGCAUGGCACUGUAAUUCAGGAAGAG	*In vitro* transcription	JRYO01000185.1	PFS in blue
tgRNA	*KRAS* WT (70 nt)	CUGCUGAAAAUGACUGAAUAUAAACUUGUGGUAGUUGGAGCUG**G**UGGCGUAGGCAAGAGUGCCUUGACGA	*In vitro* transcription	NM_033360.4	PFS in blue; SNV position in bold text
tgRNA	*KRAS* WT -1 mismatch (70 nt)	CUGCUGAAAAUGACUGAAUAUAAACUUGUGGUAGUUGGAGCUG**G**UGCCGUAGGCAAGAGUGCCUUGACGA	*In vitro* transcription	–	PFS in blue; SNV position in bold text; (mis)match underlined
tgRNA	*KRAS* WT -1 mismatch/-2 match (70 nt)	CUGCUGAAAAUGACUGAAUAUAAACUUGUGGUAGUUGGAGCUG**G**UGCUGUAGGCAAGAGUGCCUUGACGA	*In vitro* transcription	–	PFS in blue; SNV position in bold text; (mis)match underlined
tgRNA	*KRAS* WT -1 mismatch/-3 match (70 nt)	CUGCUGAAAAUGACUGAAUAUAAACUUGUGGUAGUUGGAGCUG**G**UGCCAUAGGCAAGAGUGCCUUGACGA	*In vitro* transcription	–	PFS in blue; SNV position in bold text; (mis)match underlined
tgRNA	*KRAS* G12D (70 nt)	CUGCUGAAAAUGACUGAAUAUAAACUUGUGGUAGUUGGAGCUG**A**UGGCGUAGGCAAGAGUGCCUUGACGA	*In vitro* transcription	NM_033360.4	PFS in blue; SNV position in bold text
tgRNA	*KRAS* G12D -1 mismatch (70 nt)	CUGCUGAAAAUGACUGAAUAUAAACUUGUGGUAGUUGGAGCUG**A**UGCCGUAGGCAAGAGUGCCUUGACGA	*In vitro* transcription	–	PFS in blue; SNV position in bold text; (mis)match underlined
tgRNA	*KRAS* G12D -1 mismatch/-2 match (70 nt)	CUGCUGAAAAUGACUGAAUAUAAACUUGUGGUAGUUGGAGCUG**A**UGCUGUAGGCAAGAGUGCCUUGACGA	*In vitro* transcription	–	PFS in blue; SNV position in bold text; (mis)match underlined
tgRNA	*KRAS* G12D -1 mismatch/-3 match (70 nt)	CUGCUGAAAAUGACUGAAUAUAAACUUGUGGUAGUUGGAGCUG**A**UGCCAUAGGCAAGAGUGCCUUGACGA	*In vitro* transcription	–	PFS in blue; SNV position in bold text; (mis)match underlined
tgRNA	*BRAF* WT (70 nt)	UGAAGACCUCACAGUAAAAAUAGGUGAUUUUGGUCUAGCUACAG**U**GAAAUCUCGAUGGAGUGGGUCCCAU	*In vitro* transcription	NM_004333.6	PFS in blue; SNV position in bold text

tgRNA	*BRAF* V600E (70 nt)	UGAAGACCUCACAGUAAAAAUAGGUGAUUUUGGUCUAGCUACAG**A**GAAAUCUCGAUGGAGUGGGUCCCAU	*In vitro* transcription	NM_004333.6	PFS in blue; SNV position in bold text
tgRNA	*BRCA2* WT (70 nt)	AGUGGAACCAAAUGAUACUGAUCCAUUAGAUUCAAAUGUAGCA**A**AUCAGAAGCCCUUUGAGAGUGGAAGU	*In vitro* transcription	NM_000059.4	PFS in blue; SNV position in bold text
tgRNA	*BRCA2* N372H (70 nt)	AGUGGAACCAAAUGAUACUGAUCCAUUAGAUUCAAAUGUAGCA**C**AUCAGAAGCCCUUUGAGAGUGGAAGU	*In vitro* transcription	NM_000059.4	PFS in blue; SNV position in bold text
tgRNA	*TP53* WT (70 nt)	GGCCAUCUACAAGCAGUCACAGCACAUGACGGAGGUUGUGAGGC**G**CUGCCCCCACCAUGAGCGCUGCUCA	*In vitro* transcription	NM_000546.6	PFS in blue; SNV position in bold text
tgRNA	*TP53* WT -4 mismatch (70 nt)	GGCCAUCUACAAGCAGUCACAGCACAUGACGGAGGUUGUGAGGC**G**CUGCACCCACCAUGAGCGCUGCUCA	*In vitro* transcription	–	PFS in blue; SNV position in bold text; (mis)match underlined
tgRNA	*TP53* R175H (70 nt)	GGCCAUCUACAAGCAGUCACAGCACAUGACGGAGGUUGUGAGGC**A**CUGCCCCCACCAUGAGCGCUGCUCA	*In vitro* transcription	NM_000546.6	PFS in blue; SNV position in bold text
tgRNA	*TP53* R175H -4 mismatch (70 nt)	GGCCAUCUACAAGCAGUCACAGCACAUGACGGAGGUUGUGAGGC**A**CUGCACCCACCAUGAGCGCUGCUCA	*In vitro* transcription	–	PFS in blue; SNV position in bold text; (mis)match underlined

Table S2: Plasmids used in this study.

Name	pTU#	Description	Antibiotic resistance
pTag-Csx30	pTU683	pBR322 containing *Candidatus* “Scalindua brodae” N-terminal Twin-Strep SUMO tagged Csx30 (codon optimization)	Ampicilin
pTPR-CHAT	pTU424	pACYC containing *Candidatus* “Scalindua brodae” TPR-CHAT (codon-optimized)	Chloramphenicol
pGRAMP-CRISPR-1	pTU1023	13S-S containing *Candidatus* “Scalindua brodae” Twin-Strep SUMO tagged RAMP (codon optimization) enriched with CRISPR array CRISPR1 (five times)	Streptomycin
pGRAMP-KRAS	pTU1024	13S-S containing *Candidatus* “Scalindua brodae” Twin-Strep SUMO tagged RAMP (codon optimization) enriched with CRISPR array KRAS (five times)	Streptomycin
pGRAMP-BRAF	pTU1025	13S-S containing *Candidatus* “Scalindua brodae” Twin-Strep SUMO tagged RAMP (codon optimization) enriched with CRISPR array BRAF (four times)	Streptomycin
pGRAMP-BRCA2	pTU1026	13S-S containing *Candidatus* “Scalindua brodae” Twin-Strep SUMO tagged RAMP (codon optimization) enriched with CRISPR array BRCA2 (five times)	Streptomycin
pGRAMP-TP53	pTU1027	13S-S containing *Candidatus* “Scalindua brodae” Twin-Strep SUMO tagged RAMP (codon optimization) enriched with CRISPR array TP53 (five times)	Streptomycin

References

(1) van Beljouw, S. P. B.; Haagsma, A. C.; Rodriguez-Molina, A.; van den Berg, D. F.; Vink, J. N. A.; Brouns, S. J. J. The gRAMP CRISPR-Cas effector is an RNA endonuclease complexed with a caspase-like peptidase. *Science* **2021**, *373* (6561), 1349–1353. DOI: 10.1126/science.abk2718

(2) Schindelin, J.; Arganda-Carreras, I.; Frise, E.; Kaynig, V.; Longair, M.; Pietzsch, T.; Preibisch, S.; Rueden, C.; Saalfeld, S.; Schmid, B.; et al. Fiji: an open-source platform for biological-image analysis. *Nat Methods* **2012**, *9* (7), 676–682. DOI: 10.1038/nmeth.2019

(3) Lorenz, R.; Bernhart, S. H.; Honer Zu Siederdissen, C.; Tafer, H.; Flamm, C.; Stadler, P. F.; Hofacker, I. L. ViennaRNA Package 2.0. *Algorithms Mol Biol* **2011**, *6*, 26. DOI: 10.1186/1748-7188-6-26
